# Exploring college students’ resistance to mandatory use of sports apps: a psychological reactance theory perspective

**DOI:** 10.3389/fpsyg.2024.1366164

**Published:** 2024-07-30

**Authors:** Jian Guo

**Affiliations:** Department of Physical Education, Liaoning University of Technology, Jinzhou, China

**Keywords:** psychological reactance theory, mandatory use, sports APP, students’ resistance, partial least squares structural equation modeling

## Abstract

**Introduction:**

To improve the physical fitness of college students, numerous Chinese universities have mandated students to use sports apps for running on campus. However, this has led to widespread resistance among students.

**Methods:**

To gain a deep understanding of the potential reasons for student resistance, we have developed a conceptual model based on psychological reactance theory. Specifically, we conducted a questionnaire survey involving 449 Chinese college students, using partial least squares structural equation modeling to test and analyze the research model and its related hypotheses.

**Results:**

Our results reveal that: (1) Psychological reactance poses a potential reason for students’ resistance to mandatory use of sports apps, and it has a significant negative impact on students’ attitudes and behavioral intentions. (2) Students’ perceived threat to freedom and reactance proneness are two important antecedents of psychological reactance, which can account for 51% (*R*^2^ = 0.51) of the variance in psychological reactance. Furthermore, the results indicate that students’ reactance proneness has a positive impact on perceived threats to freedom. (3) The mandatory use of sports apps leads students to have two different conditions: mandated-acceptance and mandated-rejection, both of which have a positive impact on the antecedents of psychological reactance.

**Discussion:**

These findings provide insights into the psychological processes underlying students’ resistance to mandatory use of sports apps, facilitating the application of sports apps in intervention measures that improve health and fitness. Furthermore, this study is the first to apply psychological reactance theory to mandatory exercise behavior, contributing to the reactance literature.

## 1 Introduction

Physical inactivity is the fourth leading risk factor for non-communicable diseases (WHO, 2009). Notably, among college students, physical activity declines significantly during the transition from high school to university (Bray and Born, 2004; Barton-Weston et al., 2021). Therefore, to increase physical activity among college students, numerous Chinese universities have implemented interventions mandating students to use designated sports apps (e.g., Sports World Campus app, Trail Run app, and Forked Campus app) for running on campus (hereafter referred to as “campus running”).^1^ Among them, the “Sports World Campus” app, as the most widely used sports app for campus running, has been applied in nearly 500 universities in 26 provinces and cities in China (Wang and Pan, 2020).

Campus running is a significant measure to curb the decline in physical fitness among Chinese college students, and its effectiveness has received widespread recognition for improving students’ physical fitness and cultivating exercise habits during their academic years (Yang et al., 2019; Peng and Li, 2021). However, a stringent examination of the broader implications of such programs reveals complexities in their acceptance. Drawing from James (1890), the bodily self-constitutes one of the two facets of the self, serving as a prerequisite for both agency and ownership (Gallese and Sinigaglia, 2009). This essential aspect of self may be compromised, when students are compelled to participate rather than choosing to do so willingly. Consequences of this may include impacts on their sense of agency as action initiators and their ownership over their bodily experiences. Moreover, the focus on the bodily self is a significant component of individual self-perception. Concentrating attention on the bodily self can predispose individuals to avoid loss (Sebri et al., 2021), and mandatory use of sports apps essentially forces this attention, potentially resulting in loss aversion behaviors associated with psychological reactance. Such dynamics could lead to decision-making that contradicts long-term benefits, such as widespread resistance, negative emotions, unfavorable evaluation of the campus running app, and even cheating behaviors, exemplified by hiring other students to complete their running tasks for them (Kang, 2016; Wang and Pan, 2020; Peng and Li, 2021). Furthermore, research (Wang and Pan, 2020) has indicated that if the psychological resistance of students to campus running is not addressed, even if a certain degree of short-term exercise intervention effect is achieved, it is not conducive to cultivating long-term exercise habits. Therefore, in-depth research is necessary on the potential reasons behind students’ resistance to campus running to further enhance sports apps’ positive role on students’ physical fitness. Nevertheless, existing research in this area remains relatively scarce.

Most of the existing literature focuses on the positive effects of campus running on college students’ physical health and exercise habits (Yang et al., 2019; Peng and Li, 2021), as well as the factors that influence college students’ acceptance and use of sports apps in mandatory situations (Guo, 2022). However, the negative impacts of campus running are mostly reported on social media and news reports, with a dearth of systematic scientific research. Limited research indicates that the mandatory nature of campus running leads to students exhibiting widespread and strong psychological resistance (Wang and Pan, 2020). However, empirical research based on theoretical foundations regarding the psychological mechanisms underlying this resistance is still lacking.

Psychological Reactance Theory (PRT) will provide a crucial theoretical perspective for this study. PRT is built on the assumption that individuals value their freedom. When people perceive a threat to their freedom, they are motivated to respond in opposing ways (Brehm and Brehm, 2013; Rosenberg and Siegel, 2018). College students, noted for their high autonomy and decision-making ability (Wang et al., 2022), may perceive mandated campus running as a threat to their freedom in choosing their extracurricular exercise methods, potentially leading to psychological reactance. Further, existing research provides ample evidence that mandatory actions amplify the experience of reactance (Reynolds-Tylus, 2019; Ball and Wozniak, 2021), affirming PRT’s relevance to this study. Analogously, PRT has featured prominently in studies lending insights into user resistance to mandatory use of information technology, such as mandatory use of self-service technologies (Wang and Lu, 2014; Feng et al., 2018), smart services (Wen et al., 2020), and electronic monitoring (Yost et al., 2018). Therefore, PRT provides a solid theoretical foundation for this study.

**Figure 1:**
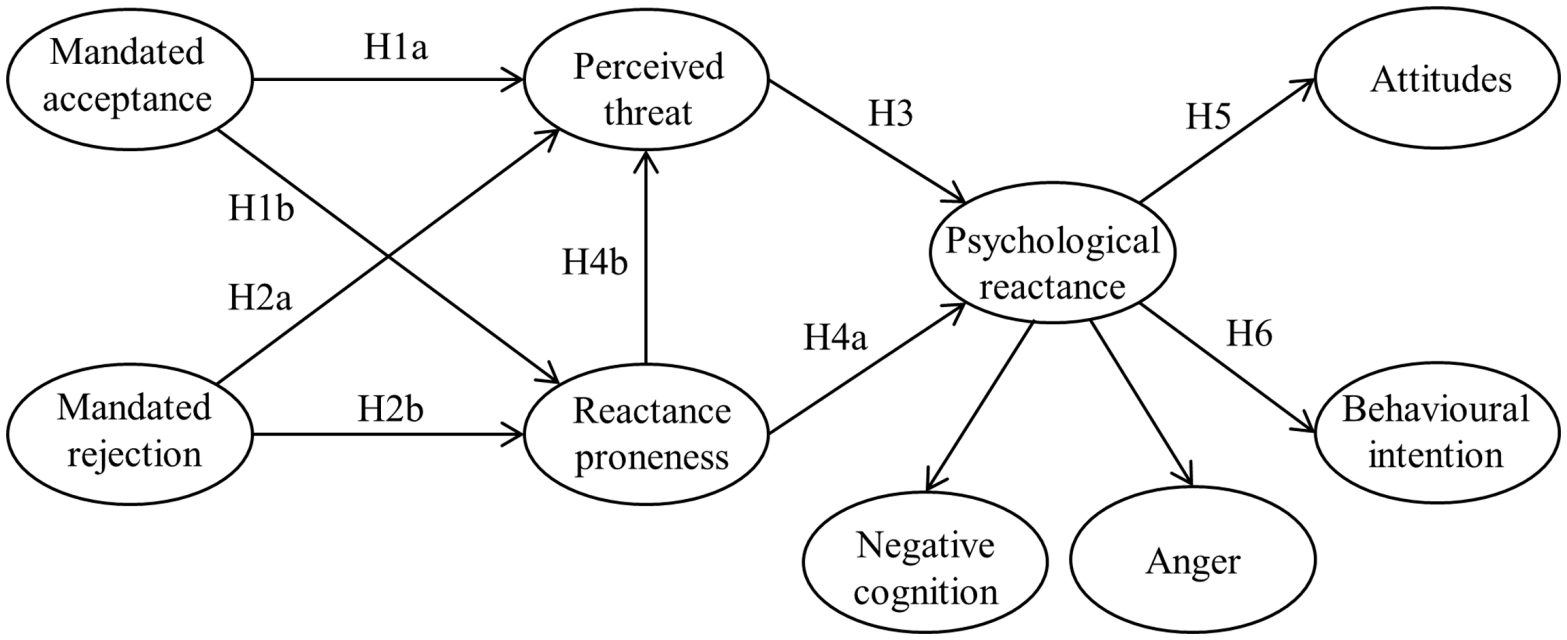
Conceptual research model.

The primary objective of the study is to explore the underlying mechanisms of student resistance to campus running from the perspective of PRT. Specifically, we develop and test a conceptual model that integrates the antecedents and outcomes of reactance against campus running. Firstly, we propose that campus running leads to two different conditions for students: mandated-acceptance and mandated-rejection. Secondly, regarding the antecedents of reactance, we examine students’ perceived freedom threat of campus running and the elicited reactance proneness. Finally, we investigate the outcomes of reactance through attitudes and behavioral intentions toward campus running. Figure 1 illustrates the conceptual model.

The main contributions of this study are as follows: (1) This study, for the first time, employs a theoretically grounded empirical research approach to investigate the underlying psychological mechanisms of student resistance to campus running. By applying a well-established theoretical framework to this novel context, we can enhance our understanding of the psychological processes underlying students’ resistance to campus running. (2) The research findings will provide valuable information to school authorities, thereby promoting the positive role of sports apps in improving college students’ physical fitness levels and cultivating exercise habits. (3) To the best of our knowledge, this is the first study to apply PRT in investigating resistance to mandatory exercise interventions, particularly those involving sports apps. Therefore, this not only extend the application of PRT to the domain of mandatory exercise behavior but also provides a novel perspective for understanding resistance in this context.

## 2 Theoretical background

### 2.1 Psychological reactance theory

The Psychological Reactance Theory (PRT) is based on the fundamental principles that individuals value freedom, choice, and autonomy. When external stimuli threaten or restrict an individual’s freedom, they are motivated to regain their freedom. This motivational state to act against the threat source and to reclaim freedom is defined as psychological reactance (Brehm and Brehm, 2013), also known as state reactance. Psychological reactance can result in negative attitudes and emotions toward the source of the threat (Silvia, 2006; Quick and Considine, 2008; Chang and Wong, 2017). Initially, Psychological reactance was considered immeasurable. However, Dillard and Shen (2005) argued that it can be assessed through both cognitive and affective aspects, specifically negative cognition and anger. When people perceive threat to their freedom, they may respond cognitively with negative evaluations of the threat source and affectively with displaying negative emotions. Additionally, academics have stressed the significance of conceptualizing the psychological reactance process as a two-step procedure that begins with a freedom threat and ends with psychological reactance (Quick et al., 2017; Rosenberg and Siegel, 2018). Thus, perceived threat to freedom is an important antecedent of psychological reactance.

Currently, TPR has been widely applied in the field of information technology to explain why users show resistance to being forced to adopt new technologies, such as self-service technology (Reinders et al., 2008; Wang and Lu, 2014; Feng et al., 2018), smart services (Wen et al., 2020), and online advertising (McCoy et al., 2017; Youn and Kim, 2019). Although this theory demonstrates strong applicability in mandatory situations, few studies have applied it to mandatory exercise behaviors.

### 2.2 Reactance proneness

Although psychological reactance was initially described as a motivational state, Brehm and Brehm (2013) proposed the existence of stable individual differences in reactance proneness. It represents the dispositional propensity to experience reactance across situations. This reactance proneness is also described as strait reactance (Moreira et al., 2021). Prior studies indicate that individuals with high reactance proneness are more sensitive to threats to their freedom (Chartrand et al., 2007), and are more reactance to influence attempts (Dillard and Shen, 2005). Given that younger individuals, particularly older adolescents, tend to exhibit the highest reactance proneness (Hong et al., 1994; Moreira et al., 2021), we incorporate reactance proneness into the conceptual model.

In the field of health communication, researchers have paid adequate attention to reactance proneness, and identified it to be an important antecedent of psychological reactance (Dillard and Shen, 2005). Considerable research has shown that people with high reactance proneness are more likely to engage in risky health behaviors (e.g., smoking, and risky sexual behaviors) and are more resistant to persuasive attempts (Van Petegem et al., 2015; Quick et al., 2017; Reynolds-Tylus, 2019). Not surprisingly, given the characteristics of trait-reactant individuals, health communication researchers consider segmenting audiences based on this personality profile a fruitful endeavor (LaVoie et al., 2016; Quick et al., 2017). However, existing research on mandatory behaviors has focused mainly on the impact of perceived freedom threat on psychological reactance (Wang and Lu, 2014; Feng et al., 2018; Wen et al., 2020; Contzen et al., 2021) while overlooking the significant role of reactance proneness. Therefore, we incorporate reactance proneness into research, which can enrich the research literature on reactance proneness in the field of mandatory behaviors.

## 3 Theoretical model and development of hypotheses

We developed a conceptual model based on PRT to explore the psychological mechanisms behind students’ resistance to the mandatory use of sports apps intervention measures, as shown in Figure 1.

### 3.1 Mandated-acceptance and mandated-rejection

We propose that the campus running leads to two different conditions for students: one is the *mandated-acceptance*, that is, students are mandated to exert additional effort to adapt to the campus running, which is triggered by the act of mandating students to accept the campus running. For instance, students with poorer physical fitness may need to make extra efforts to complete the running task, which could intrude on their bodily experiences and directly conflict with their bodily self, thus leading to their perceived threat to freedom. The other is *mandated-rejection*, that is, students are mandated to reject established lifestyles and habits to adapt to the campus running, which is triggered by the act of mandating students to reject certain extracurricular behaviors. For example, students accustomed to using mobile devices for entertainment in dormitories may need to reject their original sedentary behavior to complete the running task. This mandatory action disrupts the students’ conventional use of technology during leisure time - which is crucial to their sense of self in private spaces - thereby triggering distress and perceived loss of freedom in managing their bodily activities. Furthermore, research by Wang and Lu (2014) and Feng et al. (2018) on forcing customers to adopt self-service systems has demonstrated that mandated-acceptance and mandated-rejection are two different conditions caused by mandatory behaviors, both of which positively affect the individual’s perceived threat to freedom. Thus, we propose:

*H*1a: Mandated-acceptance positively affects students’ perceived threat to freedom.

*H*2a: Mandated-rejection positively affects students’ perceived threat to freedom.

The reactance proneness reflects individual differences in reactions to freedom-threatening stimuli (Brehm and Brehm, 2013). Both mandated-acceptance and mandated-rejection impose external pressures on students’ lifestyles, requiring them to alter their established habits or adapt to activities unfitted to their preferences. Such impositions directly infringe on their bodily experiences and personal freedom, thus evoking reactance proneness. Moreover, given that individuals have different needs for autonomy and freedom, students will perceive different degrees of freedom-threatening stimuli under these two conditions. Consequently, the reactance proneness elicited by these two conditions will also differ. We argue that the stronger the students’ experiences of these two conditions, the higher the reactance proneness elicited. Therefore, we propose:

*H*1b: Mandated-acceptance positively affects reactance proneness.

*H*2b: Mandated-rejection positively affects reactance proneness.

### 3.2 Antecedents of psychological reactance

According to PRT, a crucial component to elicit psychological reactance is the threat to freedom (Brehm and Brehm, 2013; Li et al., 2023), typically seen as an antecedent of this psychological state (Dillard and Shen, 2005; Rosenberg and Siegel, 2018). Additionally, research indicates that different types and intensities of threats to freedom can result in varying degrees of reactance. The greater the perceived threat to freedom, the stronger the psychological reactance (Reynolds-Tylus, 2019; Ball and Wozniak, 2021). Furthermore, James (2007) posits that the bodily self-constitutes the core of personal identity. Consequently, any threats to our bodily autonomy are perceived as direct threats to our core identity. When external restrictions are placed on freedom of action or the expression of our bodily self, individuals might perceive a direct threat to their core identity, thereby triggering psychological reactance as a defensive mechanism. In the context of this study, college students are mandated to use a specific sport app for running exercise, and their exercise performance is monitored. Therefore, this may lead students to perceive a threat to their extracurricular exercise autonomy or behavioral freedom, thereby eliciting psychological reactance. Hence, we propose:

*H*3: Perceived threat to freedom positively affects psychological reactance.

Apart from perceived threats to freedom, reactance proneness is another important antecedent of psychological reactance. Reactance proneness reflects an individual’s dispositional propensity to experience reactance, influencing both their sensitivity to threats to their freedom and their reactance to influence attempts (Dillard and Shen, 2005; Chartrand et al., 2007). Therefore, individuals with high reactance proneness not only perceive greater threats to their freedom but also display an intensified response of psychological reactance to such threats (Moreira et al., 2021). Numerous studies have demonstrated that an individual’s reactance proneness can have a significant positive impact on their psychological reactance (Dillard and Shen, 2005; Lowry and Moody, 2014; Van Petegem et al., 2015; Yost et al., 2018). Furthermore, Yost et al. (2018) further elaborate that individuals with high reactance proneness are more sensitive to threatening stimuli to freedom, leading them to perceive greater threats to freedom. Parallels can be drawn from health communication where it was found that this group would experience greater threats to freedom when confronted with graphic cigarette warning labels and sexual health messages (LaVoie et al., 2016; Richards and Larsen, 2016). Therefore, in the context of this study, students with high reactance proneness may not only perceive a greater threat to their extracurricular behavioral freedom, but they may also show greater psychological reactance. Based on this, we propose:

*H*4a: Reactance proneness positively affects psychological reactance.

*H*4b: Reactance proneness positively affects perceived threat to freedom.

### 3.3 Outcomes of psychological reactance

According to PRT, individuals generate psychological reactance when their freedom of choice or autonomy is hindered or threatened, typically responding with opposition or resistance to restore these freedoms (Brehm and Brehm, 2013). This psychological reactance often leads to a dislike of rules or demands, resulting in a negative attitude toward them. Due to the existence of psychological reactance, people are more inclined to stick to their original positions and behavioral patterns, reducing their willingness to undertake actions perceived as infringing on their freedom. Additionally, several studies on the mandatory use of information technology have demonstrated that psychological reactance can lead to individuals’ negative attitudes toward mandatory behavior and decreased behavioral intentions (Reinders et al., 2008; Wang and Lu, 2014; Feng et al., 2018). Similarly, in the context of this study, students’ experience of psychological reactance may negatively impact their attitudes and intentions toward campus running. Therefore, we propose:

*H*5: Psychological reactance negatively affects students’ attitudes toward campus running.

*H*6: Psychological reactance negatively affects students’ behavioral intentions toward campus running.

## 4 Methods

### 4.1 Participants

The participants in this study were all first-and second-year students from a university located in Liaoning Province, China. This university has been at the forefront of implementing mandatory use of sports apps intervention and has implemented this intervention for five consecutive years. The Physical Education Department of this university has established a comprehensive system for organizing campus running. Therefore, participants have gained extensive and consistent experiences and perceptions regarding the campus running, making their questionnaire feedback representative (Guo, 2022). This university mandates all freshmen and sophomores to participate in campus running every semester. Therefore, the data from the survey of freshmen and sophomores who are undergoing campus running are authentic.

The recruitment of participants was transparent, with individuals voluntarily participating after understanding the research objectives and procedures. Recruitment efforts were jointly conducted by members of the research team and trained interviewers. These interviewers disseminated questionnaires across various campus locations, including classrooms, dormitories, and dining halls, where they introduced the purpose, procedures, voluntary nature, and confidentiality of information related to the study to potential participants.

The inclusion criteria for participants were: freshmen and sophomores, physically healthy, without any disability that would hinder their participation in the required campus running, voluntarily participating in the study, and signing an informed consent form in accordance with the Declaration of Helsinki. Exclusion criteria included participants who failed to completely fill out the questionnaire, provided responses clearly inconsistent with actual circumstances or containing logical errors, or refused to sign the informed consent form.

### 4.2 Data collection

This study aimed to investigate the phenomenon of mandatory use, rather than a descriptive presentation of the population. The structural equation model methodology allows the use of non-probability reduced samples (Hair et al., 2014). Hence, a non-probabilistic sampling technique was employed. The surveyors distributed paper questionnaires at various locations on campus (e.g., classrooms and cafeteria entrances). The questionnaires were distributed and collected on-site from April 20th to 27th, 2023. A total of 500 questionnaires were distributed and 51 incomplete or non-compliant questionnaires were excluded. Ultimately, 449 valid questionnaires were considered, with an effective recovery rate of 89.80%.

To ensure the accuracy and completeness of the questionnaire data, the researchers thoroughly examined the contents of each questionnaire after collection. Any questionnaires that did not meet the inclusion criteria or contained missed key items were excluded from the analysis.

### 4.3 Measures

To ensure the validity of the scales, we utilized existing scales from the literature and made appropriate adaptations based on the research background and purpose. The items are as shown in Supplementary Appendix A.

#### 4.3.1 Mandated-acceptance and mandated-rejection

Drawing on the studies of Wang and Lu (2014) and Feng et al. (2018), we measured the extent to which students were mandated to accept campus running using the item, “The school mandated me to make extra efforts to adapt to mandatory campus running.” The degree to which students were mandated to reject certain extracurricular behaviors was assessed with the item, “The school mandated me to reject certain extracurricular behaviors to adapt to mandatory campus running.”

#### 4.3.2 Perceived threat to freedom

Perceived threat to freedom was measured using four items adapted from Dillard and Shen (2005) and Contzen et al. (2021), including the school “tried to manipulate my extracurricular exercise style,” “tried to pressure me to engage in campus running,” “tried to pressure me to engage in the campus running,” and “the campus running threaten my freedom to choose extracurricular behaviors.”

#### 4.3.3 Reactance proneness

Reactance proneness was assessed using four items from the Hong and Page’s scale (Hong and Faedda, 1996), including “I become frustrated when I am unable to make free and independent decisions,” “I consider advice from others to be an intrusion,” “Regulations trigger a sense of resistance in me” and “I resist the attempts of others to influence me.” This scale is frequently used to measure reactance proneness and is known for its excellent reliability (Dillard and Shen, 2005; Van Petegem et al., 2015).

#### 4.3.4 Psychological reactance

Researchers (Dillard and Shen, 2005; Rains, 2013; Feng et al., 2018) have argued that psychological reactance should be considered as a latent variable comprising cognition and affect, specifically negative cognition and anger (Quick and Considine, 2008; Quick et al., 2017). Research results in non-Western cultures still support this view (Quick and Kim, 2009). We followed this suggestion and adapted the measurement items for negative cognition and anger from Dillard and Shen (2005), Wang and Lu (2014), and Feng et al. (2018). Negative cognition was measured by four items assessing the extent to which students consider the school “ignored student’ rights to choose extracurricular exercise methods,” “did not provide enough exercise options for students,” “failed to satisfy students’ demands for extracurricular exercise” and “did not provide enough exercise facilities for students.” Anger was measured by four items assessing the extent to which students felt the “annoyed,” “unhappy,” “uncomfortable,” and “angry” about the mandated campus running.

#### 4.3.5 Attitudes and behavioral intentions

Students’ attitudes and behavioral intentions toward campus running were adapted from Wang and Lu (2014) and Feng et al. (2018). Attitude was measured through four items, including that campus running is “helpful” and “necessary” for health improvement, and it is “good” and “interesting” as an extracurricular exercise method. Behavioral intention was measured by one item, which is “I intend to use campus running as an extracurricular exercise.”

All items were assessed using a 5-point Likert-type scale, ranging from 1 (strongly disagree) to 5 (strongly agree). The specific items can be found in Supplementary Appendix A. Additionally, since all the scales were derived from English literature, a back-translation method was employed iteratively and collaboratively to ensure accurate translation of the items (Brislin, 1970; Douglas and Craig, 2007). To ensure the readability and validity of the scale, the questionnaire was pilot tested by 40 respondents. Based on the results, we made minor adjustments to the exact wording to make the statements clear.

### 4.4 Data analysis

We utilized Smart PLS 3.3.3 software and employed the second-generation statistical technique of partial least squares structural equation modeling (PLS-SEM) for model assessment. Compared to covariance-based structural equation modeling (CB-SEM), PLS-SEM is known for its robustness in exploring, predicting, and developing theories, as well as testing models with complex causal relationships and indirect effects (Hair et al., 2019). Given that the model in this study includes second-order structures, multiple latent variables, and multiple intermediate paths, PLS-SEM is the more suitable choice. Moreover, the repeated indicator approach was adopted to model the paths of the first-order constructs to misfit and obtain latent variable scores and fit for the first-order constructs (Hair et al., 2014; Li et al., 2023).

## 5 Results

### 5.1 Participant demographics

In the participant pool of this study, there were 245 males (54.57%) and 204 females (45.43%). The academic representation was balanced, comprising 239 first-year students (53.23%) and 210 s-year students (46.77%). The average age of participants was 19.39 years (*SD* = ±1.137), indicating a relatively homogeneous age group.

### 5.2 Measurement model assessment

We first deleted items with low loadings to refine the measurement model. Retained items are shown in Supplementary Appendix A. Subsequently, we assessed the reliability and validity of the measurement model. As shown in Table 1, the outer loadings of all items (ranging from 0.77 to 0.90) exceeded the threshold of 0.708. The Cronbach’s Alpha values (ranging from 0.72 to 0.88) and composite reliability (CR) values (ranging from 0.84 to 0.92) of all first-order constructs exceeded the threshold of 0.70. These results indicate favorable internal consistency and reliability of the measurement model. The average variance extracted (AVE) values of all first-order constructs (ranging from 0.64 to 0.73) exceeded the minimum requirement of 0.50, demonstrating sound convergent validity.

**Table 1:** The assessment of measurement model for constructs.

Constructs	Items	*α*	CR	AVE	Std loadings
1. Perceived threat	4	0.86	0.91	0.70	PT1 (0.82) PT2 (0.85) PT3 (0.81) PT4 (0.87)
3. Reactance proneness	3	0.72	0.84	0.64	RP2 (0.82) RP3 (0.77) RP4 (0.80)
3. Negative cognition	4	0.82	0.88	0.65	NC1 (0.77) NC2 (0.84) NC3 (0.82) NC4 (0.81)
4. Anger	4	0.88	0.92	0.73	AN1 (0.78) AN2 (0.90) AN3 (0.90) AN4 (0.84)
5. Attitudes	4	0.87	0.91	0.72	AT1 (0.85) AT2 (0.86) AT3 (0.81) AT4 (0.89)

α, Cronbach’s alpha; CR, composite reliability; AVE, average variance extracted.

In addition, the Fornell-Larcker criterion and cross-loadings were employed to assess the discriminant validity. As shown in Table 2, the correlation between any two constructs was consistently smaller than the square root of the AVE of the constructs. Additionally, each indicator’s loading exceeded its cross-loadings with other latent variables (see Supplementary Appendix B for details). These results indicate that the measurement model has satisfactory discriminant validity (Hair et al., 2014).

**Table 2:** Correlations of latent variables and evidence of discriminant validity.

Constructs	1	2	3	4	5
1. Perceived threat	**0.84**				
3. Reactance proneness	0.56	**0.80**			
3. Negative cognition	0.67	0.56	**0.81**		
4. Anger	0.54	0.43	0.57	**0.86**	
5. Attitudes	−0.62	−0.53	−0.61	−0.50	**0.85**

Bolded diagonal elements are the square root of AVE. These values should exceed inter-construct correlations (off-diagonal elements) for adequate discriminant validity.

Two techniques were employed to evaluate whether common method bias threatened the validity of the study results. The first approach used was Harman’s single-factor test (Podsakoff et al., 2003). This test involved subjecting all reflective items to both principal axis factoring and principal component factoring. In both cases, multiple factors emerged, and the variance explained by the largest factor was 40.56 and 42.31%, respectively. Both results are below the critical value of 50%. The second approach was the marker variable approach (Rönkkö and Jukka, 2011). Initially, a marker variable was identified. The marker variable was not included in the research model and had no explicit theoretical influence on the constructs in the research model. The correlation between the marker variable and research variables must be caused by the method. In this study, blue attitude served as the marker variable and was assumed to be theoretically unrelated to other variables in the study (Simmering et al., 2014). The results demonstrated that the path coefficients in the model remained virtually unchanged, with only slight variations (see Supplementary Appendix C). These findings indicate that common method bias is unlikely to distort the results of this study.

### 5.3 Structural model and hypothesis testing

**Figure 2:**
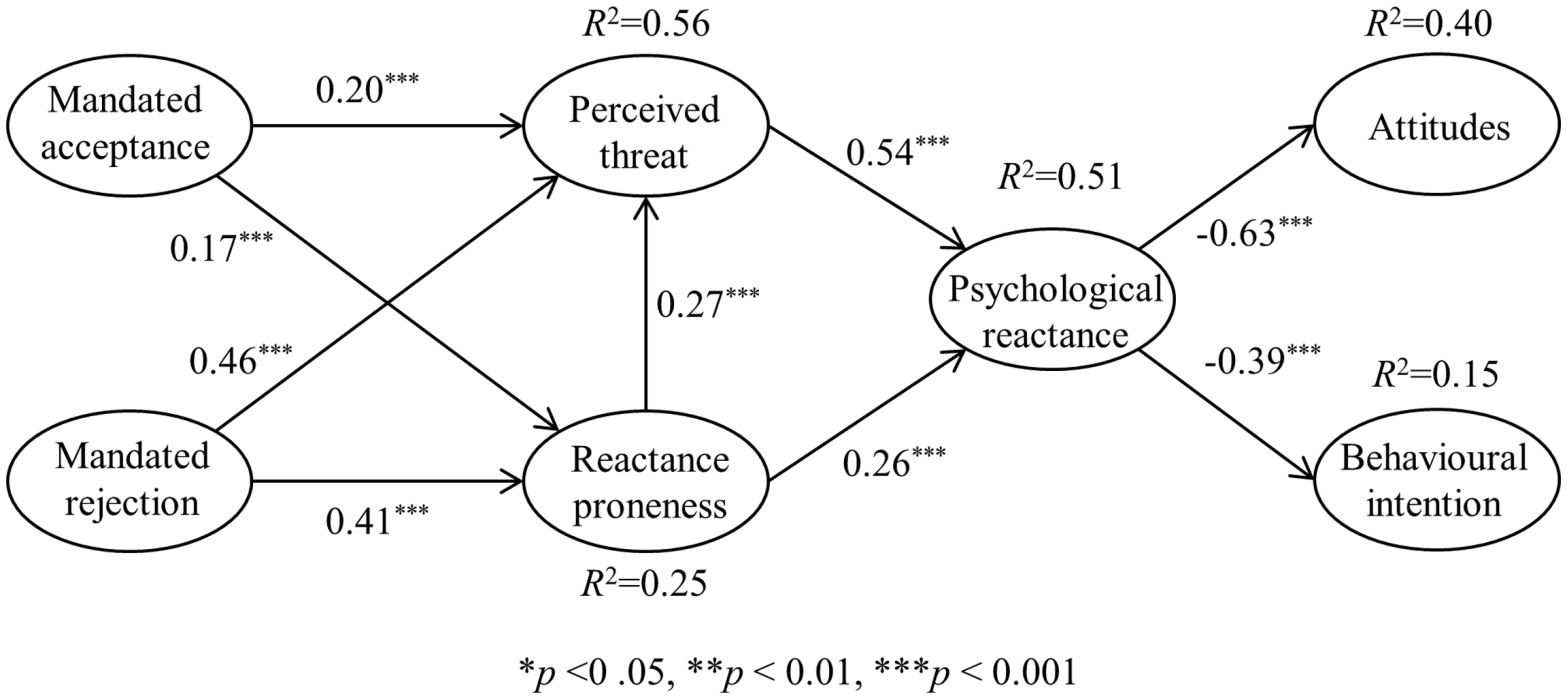
Results of the research model.

After determining the validity of the measurement model, the structural model was estimated. The bootstrapping re-sampling method (Chin, 1998) with 5,000 re-samples was used to assess the significance levels of path coefficients. The research hypothesis was considered supported when the *T*-value exceeded 1.96 (two-tailed test) and the *p* value was less than 0.05. The *R*^2^-values indicate the percentage of variance explained in the dependent variables. As illustrated in Figure 2, the model accounts for 56% of the variance in perceived threat to freedom, 25% in reactance proneness, 51% in psychological reactance, 40% in attitudes, and 15% in behavioral intention. Along with *R*^2^-values, effect size (*f*^2^) is used to determine whether a specific independent variable has a substantive impact on a dependent variable. According to Cohen (1988) guideline, the results indicate that the *f*^2^-values for the supported hypotheses are acceptable (Table 3). According to Table 3, all research hypotheses are supported. Specifically, mandated-acceptance and mandated-rejection have significant positive impacts on perceived threat to freedom (*β* = 0.20, *p* < 0.001; *β* = 0.46, *p* < 0.001) and reactance proneness (*β* = 0.17, *p* < 0.001; *β* = 0.41, *p* < 0.001), thereby supporting hypotheses H1-H2. Perceived threat to freedom and reactance proneness have significant positive impacts on psychological reactance (*β* = 0.54, *p* < 0.001; *β* = 0.26, *p* < 0.001), and reactance proneness has a significant positive impact on perceived threat to freedom (*β* = 0.27, *p* < 0.001), thereby supporting hypotheses H3-H4. Moreover, psychological reactance has a significant negative impact on attitudes (*β* = −0.63, *p* < 0.001) and behavioral intentions (*β* = −0.39, *p* < 0.001), supporting hypotheses H5-H6 (Figure 2).

**Table 3:** Path coefficients and hypotheses testing.

Hypotheses	Relationship	Path coefficient	t-statistic	*f* ^2^	Decision
H1a	MA → PT	0.20	3.47^***^	0.07	Supported
H1b	MA → RP	0.17	3.03^***^	0.03	Supported
H2a	MR → PT	0.46	6.34^***^	0.34	Supported
H2b	MR → RP	0.41	7.13^***^	0.18	Supported
H3	PT → PR	0.54	8.09^***^	0.41	Supported
H4a	RP → PR	0.26	3.82^***^	0.09	Supported
H4b	RP → PT	0.27	3.61^***^	0.13	Supported
H5	PR → AT	−0.63	17.10^***^	0.66	Supported
H6	PR → BI	−0.39	7.53^***^	0.18	Supported

MA, mandated-acceptance; MR, mandated-rejection; PT, perceived threat; RP, reactance proneness; PR, psychological reactance; AT, attitudes; BI, behavioral intention. ^***^p < 0.001.

## 6 Discussion

To reduce sedentary behavior and promote physical activity among college students, numerous Chinese universities have mandated students to use the designated sports apps for running on campus (referred to as “campus running”). However, the campus running has elicited widespread resistance from students. To explore the reasons why students resist campus running, we established a conceptual model from the perspective of PRT to explain the underlying mechanisms, and obtained several important findings.

Firstly, we propose that campus running elicits two different conditions among students: mandated-acceptance and mandated-rejection. The results confirm the significant positive impact of both conditions on the antecedents (perceived threat to freedom and reactance proneness) of psychological reactance, with mandated-rejection having a greater impact than mandated-acceptance. For students who are mandated-acceptance, despite exhibiting a certain degree of reluctance or reservation, they still make extra efforts to comply with the campus running requirements and complete the running tasks, that is, they comply reluctantly with the mandatory intervention measures (Wang and Pan, 2020). This suggests that students who are mandated-acceptance may have some degree of compliance mentality. Thus, the impact of antecedents of psychological reactance is relatively minor. For students who are mandated-rejection, they face the demand to relinquish their established habits and lifestyles to conform to campus running requirements. This directly challenges their control and management over their bodily self. According to the research of Sebri et al. (2021), focusing on the bodily self can enhance interoception promoted an aversion to losses. Under conditions of mandated-rejection, students’ bodily autonomy is limited, threatening their control over their schedules. Confronted with the potential “loss” of behavioral freedom, their perception of such losses is intensified, further diminishing their sense of control over decision-making and triggering fears about autonomy (Olison and Roloff, 2012; Cserdi and Kenesei, 2021). This amplifies the impact of mandated-rejection on the antecedents of psychological reactance. Therefore, we propose that the impact on the two antecedents of psychological reactance comes more from the perception of decision control and less from the perception of effort.

Secondly, the findings show that perceived threat to freedom and reactance proneness as antecedents both have significant positive impacts on psychological reactance, explaining 51% (*R*^2^ = 0.51) of the variance in psychological reactance. According to PRT, the perceived threat to freedom is an important prerequisite for psychological reactance, and numerous existing studies have confirmed the positive impact of perceived threat to freedom on psychological reactance (Dillard and Shen, 2005; Wang and Lu, 2014; Feng et al., 2018; Ball and Wozniak, 2021). However, in the realm of mandatory behavior studies, the profound impact of reactance proneness on psychological reactance has received less scholarly attention. Our study fills this gap by demonstrating that reactance proneness has a dual significant positive impact, not only on psychological reactance but also on perceived threat to freedom. This finding aligns with the research results from the health communication domain, where individuals with high levels of reactance proneness are more susceptible to perceiving their autonomy as threatened by persuasive messages (Richards and Larsen, 2016; Quick et al., 2017; Reynolds-Tylus, 2019). The heightened sensitivity to autonomy restrictions among individuals with high reactance proneness may stem from their heightened self-focused attention. Carver (1977) posits that individuals may perceive a greater threat to their freedom and exhibit stronger psychological reactance under high than under low self-focus. Moreover, James (1890) distinguishes the self-concept into the bodily self and the narrative self. In the context of this study, the bodily self, as power for action (Gallese and Sinigaglia, 2009), may be a focal point for self-focused attention. Individuals with high reactance proneness not only perceive that mandatory campus running directly restricting their bodily freedom (related to the bodily self), but also perceive them as disruptions to the coherence of their personal narratives (related to the narrative self). Therefore, individuals with high reactance proneness react more strongly to the same stimuli and are more sensitive to restrictions on their autonomy, thereby increasing their likelihood of perceived threat to freedom and experiencing psychological reactance (Dillard and Shen, 2005; Yost et al., 2018). Furthermore, research has demonstrated that people with high reactance proneness are more sensitive to privacy violations (Yost et al., 2018). Since sports apps involve privacy-related information such as sports data, geographic location, and personal identity, this may increase the impact of reactance proneness on perceived threat to freedom and psychological reactance. Consequently, in the context of this study, reactance proneness is not only an important antecedent of psychological reactance, but also has an important impact on perceived threat to freedom.

Thirdly, the results of this study demonstrate that psychological reactance negatively affects students’ attitudes and behavioral intentions, indicating that students’ psychological reactance can lead to negative attitudes and decreased behavioral intentions toward campus running. This is consistent with previous research on the mandatory adoption of technology (Reinders et al., 2008; Wang and Lu, 2014; Feng et al., 2018) and in the field of health communication (Dillard and Shen, 2005; Rains, 2013; Rosenberg and Siegel, 2018). Based on PRT, when individuals perceive a restriction or threat to their personal freedom, they will attempt to restore the threatened or deprived freedom in a direct or indirect manner. However, since the behavior of directly restoring freedom is usually antisocial or counter-normative, individuals typically restore freedom in an indirect manner (Brehm and Brehm, 2013). And multiple studies on the mandatory adopt of new technology have shown that negative attitudes toward mandatory behavior and decreased behavioral intentions are the primary ways for individuals to indirectly restore freedom (Reinders et al., 2008; Wang and Lu, 2014; Feng et al., 2018). Our study further corroborates the negative impact of psychological reactance on attitudes and intentions within the context of mandatory exercise. Additionally, it should be noted that psychological reactance is a state motivated by freedom concerns and the individual’s primary motivation is to restore restricted or deprived freedom. And, Ogbanufe and Gerhart (2022) argue that the adverse outcomes of psychological reactance do not necessarily reflect an evaluation of the forced use of technology, but rather an expression of restoring freedom. Therefore, students’ resistance to campus running may not be targeted at this mandatory intervention measure, but rather a manifestation of restoring extracurricular behavior freedom driven by psychological reactance.

### 6.1 Theoretical implications

This study provides several meaningful theoretical contributions to the extant literature.

The research findings provide a profound understanding of the psychological processes related to resistance against mandatory exercise interventions, particularly the utilization of sports apps. Firstly, the study’s conceptual model delineates two distinct conditions of resistance among students: mandated-acceptance and mandated-rejection. This nuanced understanding sheds light on the complex interplay between compliance psychology and concerns about autonomy. It deepens our appreciation of the psychological mechanisms driving resistance to mandatory exercise interventions. Secondly, the study underscores the pivotal roles of perceived threat to freedom and reactance proneness as antecedents of psychological reactance. Few researchers have delved into the impact of reactance proneness on psychological reactance in the context of mandatory behavior. This study fills that research gap, enhancing our understanding of the causes of psychological reactance and contributing to the literature on individual differences in resistance behaviors. Thirdly, the study’s exploration of the negative impact of psychological reactance on attitudes and behavioral intentions deepens our understanding of the consequences of reactance in the context of mandatory exercise. This aspect adds a practical dimension to the theoretical implications by highlighting the potential downstream effects of psychological reactance on individuals’ engagement with mandatory exercise interventions.

Our study contributes substantially to the existing literature on psychological reactance. Firstly, we fill a notable research gap by applying PRT to mandatory exercise behavior, thereby expanding the scope of PRT application and deepening theoretical understanding of resistance phenomena in this domain. Secondly, our research elucidates students’ resistance to campus running intervention measures from a psychological reactance perspective. By analyzing reactance mechanisms, we uncover individuals’ psychological processes when confronted with mandatory exercise interventions, offering a novel viewpoint and theoretical support for understanding and addressing resistance behaviors in similar contexts. Lastly, our study provides empirical evidence and theoretical support for research on psychological reactance. By validating the applicability of PRT in mandatory exercise behavior, we lay the groundwork and offer insights for future research, aiding scholars in better understanding and interpreting individuals’ behaviors and attitudes when faced with various mandatory interventions.

### 6.2 Practical implications

Given that psychological reactance is a potential barrier to mandatory use of sports apps intervention measures, relevant school authorities and sports app developers must formulate appropriate strategies to alleviate students’ psychological reactance, which is essential for enhancing the effectiveness of information technology in exercise interventions.

Firstly, enhancing the multifunctionality of the campus running app to reduce students’ perceived threat to freedom. The sports app chosen by the school authorities for campus running should be designed more flexibly and diversely. This design would allow students to make appropriate choices and adjustments to exercise goals and forms based on their interests and physical conditions. And this can help improve students’ autonomy and freedom of choice in campus running, thereby reducing their perceived threat to freedom. For example, under the premise of setting a total amount of physical activity, school authorities can allow students to freely choose the types, duration, intensity, and exercise venue within the sports app. This approach can meet the diverse exercise needs and preferences of different students, enhancing their willingness and enthusiasm to participate. Furthermore, it is important to avoid mandating uniform physical activities for all students.

Secondly, strengthening the publicity and education of campus running to prevent the arousal of students’ reactance proneness. To avoid triggering students’ reactance proneness, school authorities should clearly communicate the benefits and rationales of implementing intervention measures to students and emphasize the positive impact on their health and well-being. This will help to enhance students’ voluntary participation and sense of identification with mandatory interventions. Furthermore, when communicating relevant information, school authorities should try to avoid using controlling or forceful language (e.g., “you must,” “it is impossible to deny,” and “stop the denial,” etc.) (Reynolds-Tylus, 2019). These measures contribute to minimizing or avoiding triggering students’ reactance proneness.

Finally, improving the fun of campus running to alleviate the negative impact of psychological reactance. School authorities and sports app developers can take appropriate measures to improve the fun of campus running, thereby enhancing students’ attitudes and behavioral intentions toward campus running. For example, they can strengthen the community construction within the sports app, organize running clubs, hold running competitions, set virtual medals, etc., and encourage social interaction among students in the sports community, to enhance students’ sense of participation and achievement. In addition, sports app developers can develop various incentive measures, such as exchanging running mileage for small gifts, setting up a commendation mechanism for exemplary runners, and organizing exclusive running events in collaboration with well-known brands, etc. These measures help to enhance students’ intrinsic motivation and initiative to participate in campus running, thereby effectively alleviating the negative impact of psychological reactance.

In conclusion, the above practical strategies and measures will contribute to enhancing the practical application effect of campus running and promoting the sustainable development of health technology.

### 6.3 Limitations and future research

Firstly, this study adopted a cross-sectional design and relied on self-report questionnaire, which may introduce response bias and limit causal inferences. Future studies should consider adopting prospective or longitudinal research designs to establish stronger causal relationships between variables.

Secondly, this study primarily focused on psychological factors related to reactance. However, other contextual factors such as exercise environment, health cognition, and exercise interest may also influence students’ resistance to mandatory intervention measures. Future research can explore these additional factors to gain a more comprehensive understanding of the mechanisms underlying psychological resistance in the context of mandatory exercise interventions.

Finally, this study focused on the impact of psychological reactance on attitudes and behavioral intentions, and did not observe or analyze students’ actual behavior. A large number of empirical studies have shown that intention is the strongest predictor of behavior, but when faced with threats and controls of management, people may still engage in mandatory behavior even with lower behavioral intention (Lowry and Moody, 2014). Therefore, future research needs to conduct in-depth theoretical analysis and verification on the contradiction between intention and actual behavior in mandatory situations.

## 7 Conclusion

The present study provides valuable insights into the potential reasons for students’ resistance to mandatory use of sports apps intervention measures, utilizing the framework of psychological reactance theory. The findings highlight that psychological reactance is the primary reason for students’ resistance. It can lead to students’ negative attitudes and decreased behavioral intentions. Moreover, the results indicate that perceived threat to freedom and reactance proneness are two key antecedents of psychological reactance, and reactance proneness positively influences perceived threat to freedom. The impacts on these two key antecedents come more from students’ perception of decision control, and less from students’ perception of effort. These findings can help school authorities and technology developers formulate appropriate strategies and policies to mitigate the adverse effects of mandatory interventions, thereby fostering the widespread adoption of sports apps in intervention measures to enhance individuals’ fitness and overall well-being.

## Data availability statement

The original contributions presented in the study are included in the article/Supplementary material, further inquiries can be directed to the corresponding author.

## Ethics statement

Ethical approval was not required for the studies involving humans because ethical review and approval were not required for the study in accordance with the local legislation and institutional requirements. The studies were conducted in accordance with the local legislation and institutional requirements. The participants provided their written informed consent to participate in this study.

## Author contributions

JG: Writing – review & editing, Writing – original draft, Visualization, Validation, Supervision, Software, Resources, Project administration, Methodology, Investigation, Funding acquisition, Formal analysis, Data curation, Conceptualization.

## Footnotes

^1^The campus running requires students to complete a specified running mileage (e.g., at least 100 km) and frequency (e.g., at least 50 times) per semester using the schools’ designated sports app. And, during each run, students must complete a minimum running mileage (e.g., at least 2 kilometers), check-in at designated locations (e.g., check-in at least 3 points), etc. Moreover, the students’ running data are automatically uploaded to the sports app background. Physical education teachers oversee and verify students’ completion of the running tasks. If students fail to complete the required running tasks for the semester, it may affect their final physical education grades.
