# Supplementary material for: Exploring college students’ resistance to mandatory use of sports apps: a psychological reactance theory perspective
Source: Front Psychol. 2024 Jul 30;15:1366164. doi: 10.3389/fpsyg.2024.1366164 (PMC11319264; doi:10.3389/fpsyg.2024.1366164)

**Appendix A: Questionnaire items**

|  | Item content | Mean | Std dev | Kurtosis | Skewness | Loading |
| --- | --- | --- | --- | --- | --- | --- |
| **Mandated-acceptance** | |  |  |  |  |  |
| MA1 | The school mandated me to make extra efforts to adapt to mandatory campus running. | 2.63 | 1.21 | -0.86 | 0.16 | / |
| **Mandated-rejection** | |  |  |  |  |  |
| MR1 | The school mandated me to reject certain extracurricular behaviours to adapt to mandatory campus running. | 2.47 | 1.06 | -0.36 | 0.34 | / |
| **Perceived threat** | |  |  |  |  |  |
| PT1 | The campus running threaten my freedom to choose extracurricular behaviours. | 2.47 | 1.11 | -0.34 | 0.47 | 0.82 |
| PT2 | The school tried to decide on my extracurricular exercise methods. | 2.62 | 0.99 | -0.17 | 0.16 | 0.85 |
| PT3 | The school tried to manipulate my extracurricular exercise style. | 2.65 | 0.94 | 0.09 | 0.04 | 0.81 |
| PT4 | The school tried to pressure me to engage in the campus running | 2.44 | 0.93 | -0.18 | 0.12 | 0.87 |
| **Reactance proneness** | |  |  |  |  |  |
| RP1* | I become frustrated when I am unable to make free and independent decisions. |  |  |  |  |  |
| RP2 | I consider advice from others to be an intrusion. | 2.73 | 0.95 | 0.13 | 0.09 | 0.82 |
| RP3 | Regulations trigger a sense of resistance in me. | 3.03 | 1.05 | -0.25 | -0.06 | 0.77 |
| RP4 | I resist the attempts of others to influence me. | 2.46 | 0.94 | 0.20 | 0.32 | 0.80 |
| **Negative cognition** | |  |  |  |  |  |
| NC1 | The school ignored student’ rights to choose extracurricular exercise methods. | 2.74 | 1.15 | -0.66 | 0.10 | 0.77 |
| NC2 | The school didn't provide enough exercise options for students. | 2.54 | 0.99 | -0.33 | 0.20 | 0.84 |
| NC3 | The school failed to satisfy students' demands for extracurricular exercise. | 2.61 | 1.03 | -0.18 | 0.29 | 0.82 |
| NC4 | The school didn't provide enough exercise facilities for students. | 2.40 | 0.95 | -0.07 | 0.30 | 0.81 |
| **Anger** | |  |  |  |  |  |
| AN1 | The campus running annoyed me. | 2.67 | 1.26 | -0.88 | 0.31 | 0.78 |
| AN2 | The campus running made me unhappy. | 2.37 | 1.01 | -0.44 | 0.39 | 0.90 |
| AN3 | The campus running made me angry. | 2.49 | 0.97 | 0.11 | 0.51 | 0.90 |
| AN4 | The campus running made me feel uncomfortable. | 2.30 | 1.04 | -0.10 | 0.56 | 0.84 |
| **Attitudes** | |  |  |  |  |  |
| AT1 | The campus running is very helpful for improving physical health. | 3.57 | 1.05 | -0.11 | -0.45 | 0.85 |
| AT2 | The campus running is a very good extracurricular exercise method. | 3.29 | 1.02 | -0.37 | -0.05 | 0.86 |
| AT3 | The campus running is very necessary for students. | 3.70 | 0.89 | 0.27 | -0.35 | 0.81 |
| AT4 | The campus running is a very interesting extracurricular exercise method. | 3.46 | 0.97 | 0.33 | -0.37 | 0.89 |
| **Behaviour Intention** | |  |  |  |  |  |
| BI1 | I intend to use campus running as an extracurricular exercise. | 2.94 | 1.03 | -0.13 | 0.01 | / |

Note: *Item dropped from final analysis; MA = mandated-acceptance; MR = mandated-rejection; PT = perceived threat; RP = reactance proneness; NC = negative cognition; AN = anger; AT = attitudes; BI = behaviour intention.

**Appendix B: Items loadings and cross loadings.**

|  | PT | RP | NC | AN | AT |
| --- | --- | --- | --- | --- | --- |
| PT1 | **0.82** | 0.45 | 0.57 | 0.47 | -0.52 |
| PT2 | **0.85** | 0.48 | 0.58 | 0.48 | -0.52 |
| PT3 | **0.81** | 0.41 | 0.48 | 0.39 | -0.45 |
| PT4 | **0.87** | 0.54 | 0.61 | 0.48 | -0.58 |
| RP2 | 0.42 | **0.82** | 0.44 | 0.29 | -0.37 |
| RP3 | 0.44 | **0.77** | 0.38 | 0.39 | -0.44 |
| RP4 | 0.49 | **0.80** | 0.53 | 0.36 | -0.45 |
| NC1 | 0.52 | 0.38 | **0.77** | 0.48 | -0.46 |
| NC2 | 0.55 | 0.44 | **0.84** | 0.43 | -0.47 |
| NC3 | 0.47 | 0.40 | **0.82** | 0.43 | -0.44 |
| NC4 | 0.62 | 0.59 | **0.81** | 0.49 | -0.61 |
| AN1 | 0.44 | 0.30 | 0.47 | **0.78** | -0.45 |
| AN2 | 0.49 | 0.38 | 0.49 | **0.90** | -0.43 |
| AN3 | 0.47 | 0.38 | 0.49 | **0.90** | -0.43 |
| AN4 | 0.46 | 0.41 | 0.49 | **0.84** | -0.41 |
| AT1 | -0.54 | -0.45 | -0.50 | -0.39 | **0.85** |
| AT2 | -0.55 | -0.46 | -0.54 | -0.50 | **0.86** |
| AT3 | -0.43 | -0.36 | -0.49 | -0.37 | **0.81** |
| AT4 | -0.58 | -0.52 | -0.55 | -0.43 | **0.89** |

Note: Numbers in bold = indicator’s outer loadings on the associated construct.

**Appendix C:** **Test for common method variance (marker variable)**


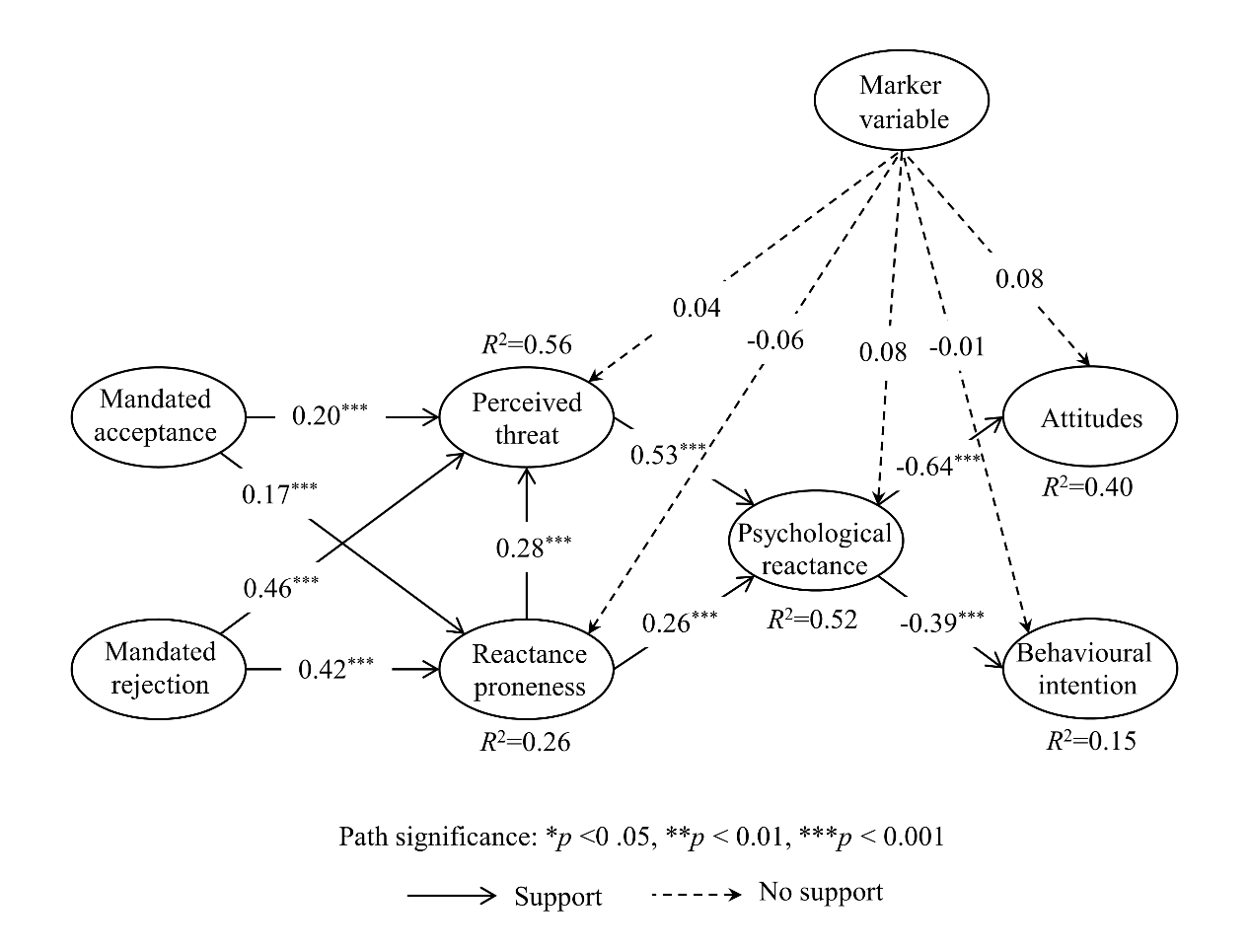

Supplement: Supplementary file 1 [file Table_1.docx]
